# Head-to-head comparison of ^18^F-FAPI and ^18^F-FDG PET/CT in staging and therapeutic management of hepatocellular carcinoma

**DOI:** 10.1186/s40644-023-00626-y

**Published:** 2023-10-30

**Authors:** Jing Zhang, Shuqin Jiang, Mengsi Li, Haibao Xue, Xi Zhong, Shuyi Li, Hao Peng, Jiuceng Liang, Zhidong Liu, Songquan Rao, Haipeng Chen, Zewen Cao, Yuanfeng Gong, Guoshuo Chen, Rusen Zhang, Linqi Zhang

**Affiliations:** 1 Department of Nuclear Medicine, Affiliated Cancer Hospital & Institute of Guangzhou Medical University, 78 Hengzhigang Road, Guangzhou, 510095 People’s Republic of China; 2 Department of Nuclear Medicine, the First Affiliated Hospital of Guangzhou Medical University, No.28 Qiaozhong Road, Guangzhou, Guangdong 510163 P. R. China; 3 Department of Radiology, the Third Affiliated Hospital of Sun Yat-Sen University, No.600, Tianhe Road, Guangzhou, Guangdong 510630 P. R. China; 4 Department of Radiology, Affiliated Cancer Hospital & Institute of Guangzhou Medical University, 78 Hengzhigang Road, Guangzhou, Guangdong 510095 P. R. China; 5 Department of Hepatobiliary Surgery, Affiliated Cancer Hospital & Institute of Guangzhou Medical University, 78 Hengzhigang Road, Guangzhou, Guangdong 510095 P. R. China; 6 Department of Interventional Medicine, Affiliated Cancer Hospital & Institute of Guangzhou Medical University, 78 Hengzhigang Road, Guangzhou, Guangdong 510095 P. R. China

**Keywords:** Hepatocellular carcinoma, Fibroblast activation protein, ^18^F-FAPI; PET, CT

## Abstract

**Background:**

Fluorine 18 (^18^F) fluorodeoxyglucose (FDG) positron emission tomography/computed tomography (PET/CT) has limitations in staging hepatocellular carcinoma (HCC). The recently introduced ^18^F-labeled fibroblast-activation protein inhibitor (FAPI) has shown promising prospects in detection of HCC lesions. This study aimed to investigate the initial staging and restaging performance of ^18^F-FAPI PET/CT compared to ^18^F-FDG PET/CT in HCC.

**Methods:**

This prospective study enrolled histologically confirmed HCC patients from March 2021 to September 2022. All patients were examined with ^18^F-FDG PET/CT and ^18^F-FAPI PET/CT within 1 week. The maximum standard uptake value (SUV_max_), tumor-to-background ratio (TBR), and diagnostic accuracy were compared between the two modalities.

**Results:**

A total of 67 patients (57 men; median age, 57 [range, 32–83] years old) were included. ^18^F-FAPI PET showed higher SUV_max_ and TBR values than ^18^F-FDG PET in the intrahepatic lesions (SUV_max_: 6.7 vs. 4.3, *P* < 0.0001; TBR: 3.9 vs. 1.7, *P* < 0.0001). In diagnostic performance, ^18^F-FAPI PET/CT had higher detection rate than ^18^F-FDG PET/CT in intrahepatic lesions [92.2% (238/258) vs 41.1% (106/258), *P* < 0.0001] and lymph node metastases [97.9% (126/129) vs 89.1% (115/129), *P* = 0.01], comparable in distant metastases [63.6% (42/66) vs 69.7% (46/66), *P* > 0.05]. ^18^F-FAPI PET/CT detected primary tumors in 16 patients with negative ^18^F-FDG, upgraded T-stages in 12 patients and identified 4 true positive findings for local recurrence than ^18^F-FDG PET, leading to planning therapy changes in 47.8% (32/67) of patients.

**Conclusions:**

^18^F-FAPI PET/CT identified more primary lesions, lymph node metastases than ^18^F-FDG PET/CT in HCC, which is helpful to improve the clinical management of HCC patients.

**Trial registration:**

Clinical Trials, NCT05485792. Registered 1 August 2022, Retrospectively registered.

**Supplementary Information:**

The online version contains supplementary material available at 10.1186/s40644-023-00626-y.

## Background

Hepatocellular carcinoma (HCC) is the most common type of primary liver cancer, and the fourth leading cause of cancer-related deaths worldwide [1]. Patients diagnosed in early stages of the disease have survival rates up to 50–70%. However, more than half of patients were diagnosed with advanced disease, and their 3-year survival rates were only 20–30% [2–4]. Therefore, early diagnosis and accurate staging for HCC patients are critical for planning therapy. Morphological imaging modalities, such as contrast computed tomography (CT) and magnetic resonance imaging (MRI), are commonly used in the diagnosis of HCC, but they are inadequate in detecting distant metastasis [5]. In addition, due to the postoperative anatomical changes, it is difficult to monitor the recurrence of HCC based on morphological imaging modalities [6]. Fluorine-18 fluorodeoxyglucose (^18^F-FDG) positron emission tomography/computed tomography (PET/CT) is an effective imaging tool for staging for many malignancies. However, ^18^F-FDG is not a useful tracer for detection of primary tumours of HCC [7]. Because of the low ^18^F-FDG uptake in well differentiated HCC and the physiological uptake in nomal liver, the detection rate of ^18^F-FDG PET/CT for primary HCC is less than 50% [8].

Fibroblast activation protein (FAP) is a serine protease that belongs to the dipeptidyl peptidase-IV (DPP-IV) family located in fbroblast membranes [9]. FAP is overexpressed in the cancer-associated fibroblasts (CAFs) of 90% of all epithelial carcinomas, including HCC [10]. Therefore, FAP-targeted radiopharmaceuticals can be considered a promising approach for the visualization of CAFs in HCC. Recently, ^68^ Ga labeled fibroblast activating protein inhibitor (^68^ Ga-FAPI) has demonstrated diagnostic value in many types of malignancies [11]. Moreover, several pilot studies with small sample size have shown ^68^ Ga-FAPI PET/CT is more sensitive than ^18^F-FDG PET/CT in detecting HCC lesions [3, 12–14]. Although ^68^ Ga-FAPI is a promising radiopharmaceutical for clinical application of malignancies, it still has some disadvantages, such as high production costs and short half-life [15–17]. ^18^F-FAPI has a longer half-life and is more widely used to meet the needs of a large number of patients, and ^18^F-FAPI is equivalent to ^68^ Ga-FAPI in detecting malignant tumors [17]. However, to our acknowledge, there is no study have explored the clinical staging value of ^18^F-FAPI PET/CT in HCC systematically.

Therefore, the aim of this head to head prospective study was to investigate whether the potential diagnostic value of ^18^F-FAPI PET/CT is superior to ^18^F-FDG PET/CT for HCC patients, and to explore the impact of ^18^F-FAPI PET/CT on the clinical therapeutic management of HCC.

## Materials and methods

### Patients

**Fig. 1:**
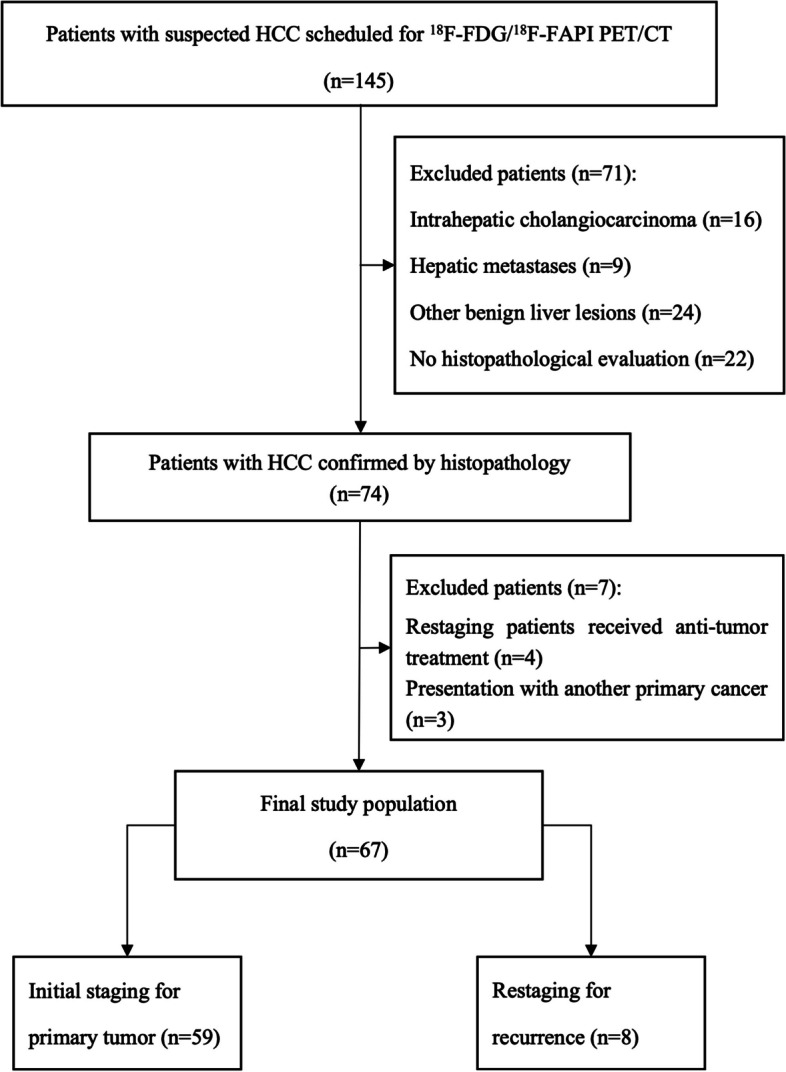
Study flowchart shows inclusion and exclusion criteria. HCC = hepatocellular carcinoma; ^18^F = fluorine 18; FAPI = fibroblast activation protein inhibitor; FDG = fluorodeoxyglucose; PET/CT = positron emission tomography/computed tomography

This prospective study was authorized by the ethics committee of Affiliated Cancer Hospital & Institute of Guangzhou Medical University (ethics committee permission No.2021-sw07; clinical trial registration: NCT05485792). From March 2021 to September 2022, A total of 145 patients with suspected HCC were considered as candidate participants consecutively. The enrolled patients met the following criteria: (i) age ≥ 18 years old; (ii) patients with suspected liver malignant lesions based on traditional diagnostic imaging (CT or MRI or ultrasound) and clinical symptoms; and (iii) patients who agreed to receive paired ^18^F-FDG PET/CT and ^18^F-FAPI PET/CT scans within one week. The exclusion criteria were as follows: (i) restaging patients who have received chemotherapy, radiation therapy, or targeted therapy within 3 months prior to scanning; (ii) patients who had another primary cancer at the time of evaluation; and (iii) unable to provide pathological findings to confirm HCC. Finally, a total of 67 patients were enrolled. The flow chart of our study was depicted in Fig. 1.

### ^18^F-FDG/^18^F-FAPI PET/CT acquisition and imaging

^18^F-FDG was automatically synthesized using a PET trace cyclotron and the ^18^F-FDG synthesizer module (Tracerlab FXF-N, GE Healthcare). The detailed methodology for radiolabeling DOTA-FAPI can be found in the Supplementary material. ^18^F-FDG and ^18^F-FAPI PET/CT were performed using a PET/CT scanner (Discovery 710, GE Healthcare, Milwaukee, WI, USA) within 1 week. The imaging preparation and parameters of ^18^F-FDG/^18^F-FAPI PET/CT was performed according to a previously reported protocol [18].

### ^18^F-FDG and ^18^F-FAPI PET/CT image analysis

All images were visually interpreted independently by four board certified nuclear medicine physicians. To reduce individual interpretation bias, ^18^F-FDG PET/CT images were reviewed by Hao Peng and Shuyi Li, and ^18^F-FAPI PET/CT images were reviewed by Shuqin Jiang and Linqi Zhang. A consensus was reached following a comprehensive discussion in cases of discrepancies.

For visual analysis, lesions were divided into primary tumor and extrahepatic organs/regions (lymph nodes and distant metastasis) based on their location. Individual lymph node was then classified into four regions, including the head and neck, thoracic (supraclavicular, mediastinal, and axillary lymph nodes), abdominal (para-aortic, porta hepatic, retroperitoneal, celiac lymph nodes), and pelvic (parailiac vessels and inguinal lymph nodes). Distant involvement such as lung, bone, peritoneal and adrenal metastasis was categorized as an individual site. A positive lesion was considered when met the following criteria [19]: (i) a focal area had abnormally elevated ^18^F-FDG or ^18^F-FAPI uptake, accompanied by the abnormal density/signal in the corresponding sites on CT/MRI; and (ii) the lesions had typical features from contrast-enhanced CT (ceCT) or ce-MRI.

For semi-quantitative assessment, a region of interest (ROI) was drawn along the entire lesion on the axial PET image or anatomical information presented by CT/MRI (lesions with low or equal tracer uptake), and the maximum standardized uptake value (SUV_max_), the diameter of each lesion and the amount of lesions per region were recorded. The tumor-to-background ratio (TBR) of each lesion was calculated by dividing the SUV_max_ of the lesion by the SUV_mean_ of the background tissue (liver background for liver lesions; mediastinal blood pool background for macrovascular invasion, lymph nodes and peritoneal lesions; lung background for lung lesions; contralateral adrenal gland background for adrenal gland lesions; and L5 background for bone lesions). The TNM stage was assigned based on the eighth edition of the American Joint Committee on Cancer staging system [20].

### Reference standard

All patients were diagnosed HCC (at least one lesion) based on histological evaluation of biopsy or surgical specimens. Due to ethical and technical issues, not all lesions are pathologically confirmed, especially for intrahepatic foci, lymph node metastasis and distant metastasis. When histopathology was unavailable for positive PET/CT findings, a combination of clinical and multimodality radiographic (including PET/CT, contrast-enhanced CT, MRI, and ultrasound) follow-up for more than 6 months was taken as the reference standard of diagnosis to validate the PET/CT findings [18]. Follow-up imaging findings that were considered malignant lesions had either progress or response to anticancer therapy in terms of reduction in size and/or number of lesions.

### Statistical analysis

Quantitative variables are presented as the median [range (minimum to maximum)], and categorical variables are presented as frequencies (percentages). The diagnostic performance for HCC of ^18^F-FDG PET and ^18^F-FAPI PET was compared using the McNemar’s test. The SUV_max_ and TBR obtained from ^18^F-FDG PET and ^18^F-FAPI PET images were compared using the paired Wilcoxon signed-rank test. All statistical tests were performed using SPSS Statistics 17.0 (SPSS Inc., Chicago, IL, USA) software. *P* < 0.05 was considered statistically significant.

## Results

### Patients characteristics

Sixty-seven patients with histological proven HCC (57 men and 10 women; median age, 57 [range, 32–83] years old) were enrolled in our study (Fig. 1). The most common etiology was hepatitis B infection (*n* = 46, 68.7%), and eighteen patients presented with cirrhosis. Fifty-nine treatment-naive patients received paired ^18^F-FDG and ^18^F-FAPI PET examinations for initial staging, and 8 patients with recurrent HCC underwent paired examinations for restaging. 26.9% (18 of 67) of patients had intrahepatic lesions invading macrovascular, and 19.4% (13 of 67) of patients were identified to have extrahepatic metastasis (6 patients with lymph node metastasis and 9 patients with distant metastasis) (Table 1).

**Table 1:** Baseline patient characteristics of the enrolled patients

Description of patients	67
Age [years, median (IQR)]	57(32–83)
M:F radio	57:10
Cirrhosis/Non-Cirrhosis	18/49
Etiology (HBV/HCV/AH)	46/1/1
Clinical biochemical testing	
AFP (> 20 ng/ml)	33
CEA (> 5U/ml)	21
CA19-9 (> 37U/ml)	9
Patient status	
Staging	59
Recurrence detection after treatment	8
Tumor number	
Solitary	32
Multifocal	35
Macrovascular invasion (Yes/No)	18/49
Tumor staging (Initial evaluation)	
T1	16
T2	13
T3	14
T4	16
Extrahepatic Lesions (N/total)	13/67
Lymph node metastasis	7
Distant metastasis	9

*M* male, *F* female, *HBV* Hepatitis B virus, *HCV* Hepatitis C virus, *AH* Alcoholic hepatitis, *IQR* interquartile range, *AFP* alpha-fetoprotein, *CEA* carcinoembryonic antigen, *CA 19–9* carbohydrate antigen 199

### ^18^F-FDG PET and ^18^F-FAPI PET in detection of intrahepatic lesions

**Fig. 2:**
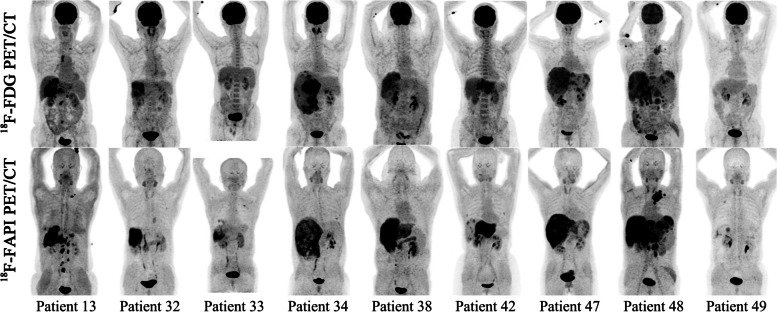
Nine representative patients with HCC underwent ^18^F-FDG & ^18^F-FAPI PET/CT imaging. ^18^F-FAPI PET/CT outperformed.^18^F-FDG PET/CT in detecting primary tumors (Patient No. 32, 33, 34, 49), intrahepatic subfoci (Patient No. 13, 38, 42, 47, 48), supraclavicular lymph node metastases (Patient No. 13, 48), retroperitoneum lymph node metastases (Patient No. 48), and comparable in detecting distant metastases (Patient No. 48)

**Fig. 3:**
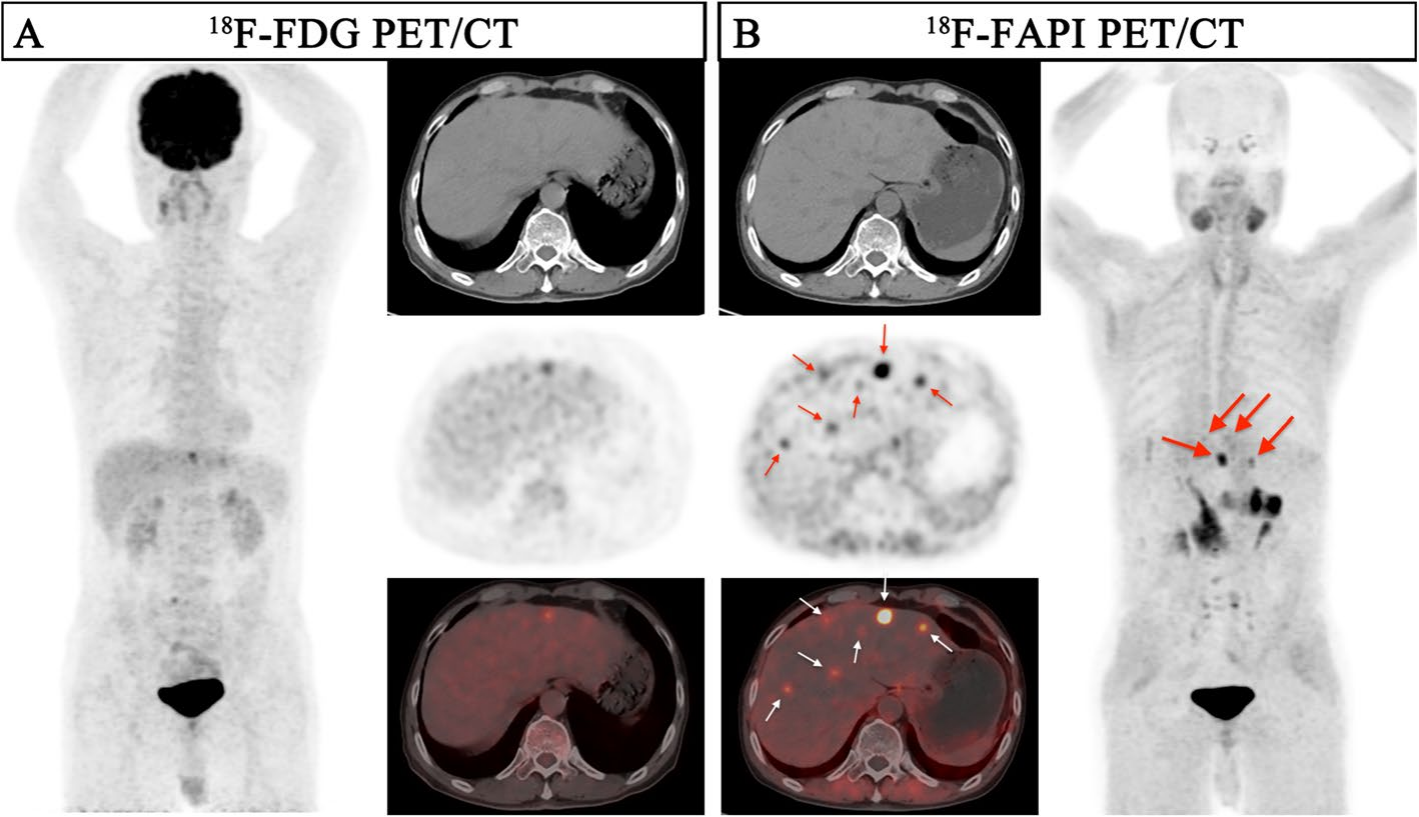
A 41-year-old male patient (Patient No. 51) with HCC (moderately differentiated) was confirmed by biopsy. ^18^F-FDG PET/CT displayed moderate uptake in the section II of the liver; However, the corresponding CT scan showed more nodules in other lobes of the liver. ^18^F-FAPI PET/CT detects greater radiotracer in primary lesions and other intrahepatic subfoci on both MIP (large arrow) and axial images (small arrow)

In the initial staging group of 59 patients (a total of 234 lesions), the detecting rate of ^18^F-FAPI PET for intrahepatic lesions is significantly higher than ^18^F-FDG PET among patients with T1-3 stages (detail in Table 2, Figs. 2 and 3) [T1: 93.8% (15/16) vs. 31.3% (5/16), *P* = 0.0006; T2: 100% (13/13) vs. 38.5% (5/13), *P* = 0.0016; T3: 100% (14/14) vs. 35.7% (5/14), *P* = 0.0006; T4: 100% (16/16) vs. 100% (16/16), *P* > 0.05], and the lesions in T2-4 stage patients were more clearly characterized by higher activity (median SUV_max_, T2: 9.9 vs. 5.3, *P* = 0.0339; T3: 10.9 vs. 5.5, *P* = 0.0085; T4: 12.9 vs. 14.5, *P* = 0.0457) and clearer boundaries (median TBR, T2: 5.0 vs. 1.7, *P* = 0.0002; T3: 6.6 vs. 2.1, *P* = 0.0001; T4: 10.2 vs. 3.1, *P* < 0.0001) in ^18^F-FAPI PET than in ^18^F-FDG PET (Supplementary Fig. 1a & b).

**Table 2:** Comparison of ^18^F-FDG PET/CT and ^18^F-FAPI PET/CT for the intrahepatic lesions of 67 patients

Description of lesions	No. of patients (No. of lesions)	^18^F-FDG PET/CT			^18^F-FAPI PET/CT			*P* value (FDG vs. FAPI)	
		Positive detection of lesions(%)	Median SUV_max_ (range)	Median TBR (range)	Positive detection of lesions(%)	Median SUV_max_ (range)	Median TBR (range)	SUV_max_	TBR
Total	67	35(52.2%)	5.3(2.3–22.5)	2.1(1.0–15.2)	66(98.5%)	9.9(0.4–24.1)	6.0(0.8–30.0)	** < 0.0001**	** < 0.0001**
Primary Tumor	59	31(53.4%)	5.6(2.3–22.5)	2.2(1.0–15.2)	58(98.3%)	9.9(0.4–24.1)	6.7(0.8–30.0)	** < 0.0001**	** < 0.0001**
T1	16	5(31.3%)	3.75(2.3–11.2)	1.5(1.0–4.3)	15(93.8%)	4.65(0.4–15.2)	3.6(0.8–27.8)	0.1305	** < 0.0001**
T2	13	5(38.5%)	5.3(2.3–22.5)	1.7(1.4–9.8)	13(100%)	9.9(4.6–15.6)	5.0(1.5–15.0)	**0.0339**	**0.0002**
T3	14	5(35.7%)	5.5(3.7–15.5)	2.1(1.3–6.4)	14(100%)	10.9(3.3–24.1)	6.6(2.6–30.0)	**0.0085**	**0.0001**
T4	16	16(100%)	14.5(4.1–18.4)	3.5(1.9–15.2)	16(100%)	12.9(2.2–22.9)	10.2(4.1–22.5)	**0.0457**	** < 0.0001**
Recurrent Tumor	8	4(50.0%)	3.6(2.4–11.8)	1.4(1.0–5.2)	8(100%)	9.8(2.9–16.8)	5.4(2.6–12.9)	0.0781	**0.0078**
Tumor Size(cm)									
Total	67(258)	106(41.1%)	4.3(1.3–22.5)	1.7(0.5–15.2)	238(92.2%)	6.7(0.4–24.1)	3.9(0.8–30.0)	** < 0.0001**	** < 0.0001**
≤ 2	NA(110)	28(25.5%)	3.7(1.3–9.8)	1.5(0.5–4.3)	97(88.1%)	5.9(1.2–18.3)	3.1(0.8–22.9)	** < 0.0001**	** < 0.0001**
> 2, ≤ 5	NA(89)	34(38.2%)	4.2(2.2–13.8)	1.6(0.8–10.3)	82(92.1%)	6.5(0.4–22.4)	3.8(0.8–30.0)	** < 0.0001**	** < 0.0001**
> 5	NA(59)	44(74.6%)	7.1(3.7–22.5)	2.5(1.3–15.2)	59(100%)	11.2(2.2–24.1)	6.6(2.6–26.1)	** < 0.0001**	** < 0.0001**
No. of lesions									
Solitary	32(32)	12(37.5%)	4.35(2.3–22.5)	1.7(1.0–9.8)	31(96.9%)	6.35(0.4–15.6)	5.15(0.8–27.8)	**0.0152**	** < 0.0001**
Multifocal	35(226)	94(41.6%)	4.3(1.3–18.4)	1.7(0.5–15.2)	207(91.6%)	6.7(1.2–24.1)	3.8(0.8–30.0)	** < 0.0001**	** < 0.0001**
Macrovascular invasion	18(18)	18(100%)	5.6(2.7–23.3)	3.5(1.4–12.3)	16(88.9%)	4.65(0.5–8.2)	2.9(1.2–4.6)	**0.007**	**0.043**

Bold fonts indicate significant difference between FDG and FAPI (*P* < 0.05)

^*18*^*F* Fluorine 18, *FDG* fluorodeoxyglucose, *FAPI* fibroblast activation protein inhibitor, *PET/CT* positron emission tomography/computed tomography, *HCC* hepatocellular carcinoma, *SUV*_*max*_ maximum standardized uptake value, *TBR* Tumor-to-background ratio, *NA* Not applicable

**Fig. 4:**
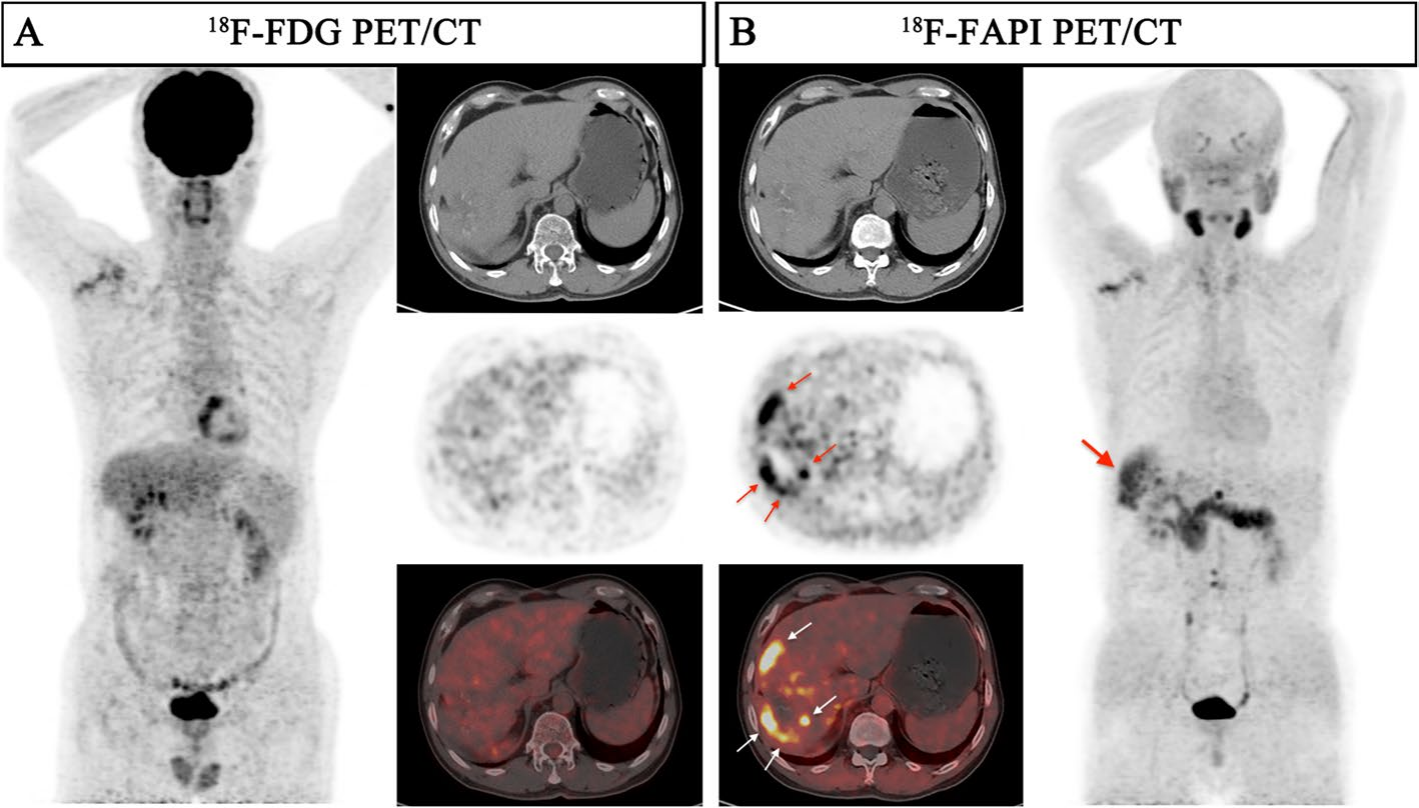
A 57-year-old male patient (Patient No. 50) with recurrent HCC (moderately differentiated) was confirmed by postoperative pathology. ^18^F-FDG PET/CT displayed no uptake in this lesion, although the corresponding CT scan showed lamellar low-density shadow in right lobe of the liver. ^18^F-FAPI PET/CT revealed intense uptake (SUV_max_ 9.0; TBR 6.0) in the recurrent lesion on both maximum intensity projection (MIP) (large arrow) and axial images (small arrow)

In the evaluation of recurrent tumors in 8 patients (a total of 24 lesions), there was no statistically significance in the sensitivity of detecting recurrent tumors between ^18^F-FAPI PET and ^18^F-FDG PET [100% (8/8) vs. 50.0% (4/8), *P* = 0.0769], while the TBR of ^18^F-FAPI PET/CT was significantly higher than that of ^18^F-FDG PET/CT for local recurrence (median TBR: 5.4 vs 1.4, *P* = 0.0078) (Table 2 and Fig. 4).

As shown in Table 2, ^18^F-FAPI PET/CT depicted 92.2% of the intrahepatic lesions (238 of 258), which was much better than 41.1% (106 of 258) of ^18^F-FDG PET/CT (*P* < 0.0001). According to tumour size, ^18^F-FAPI PET detected significantly more intrahepatic lesions than ^18^F-FDG PET among different sizes subgroups, especially in the early stage HCC (Fig. 3) [≤ 2 cm: 88.1% (97/110) vs. 25.5% (28/110), *P* < 0.0001; > 2 cm and ≤ 5 cm: 89.9% (80/89) vs. 38.2% (34/89), *P* < 0.0001; > 5 cm: 100% (59/59) vs. 74.6% (44/59), *P* < 0.0001]. Besides, there were also significant differences in ^18^F-FAPI PET and ^18^F-FDG PET uptake among different tumor size groups (all *P* < 0.0001, Supplementary Fig. 1c & d).

A total of 18 patients had macrovascular invasion (16 patients for initial staging; 2 patients for restaging). There was no statistically significance in the sensitivity of detecting macrovascular invasion between ^18^F-FAPI PET and ^18^F-FDG PET [88.9% (16/18) vs. 100% (18/18), *P* = 0.486], and the SUV_max_ and TBR of 18 paired macrovascular invading lesions on ^18^F-FAPI PET/CT images were significantly lower than on ^18^F-FDG PET/CT images (*P* = 0.007 and *P* = 0.043, respectively) (Table 2).

### ^18^F-FDG PET and ^18^F-FAPI PET for assessment of lymph node metastasis

**Fig. 5:**
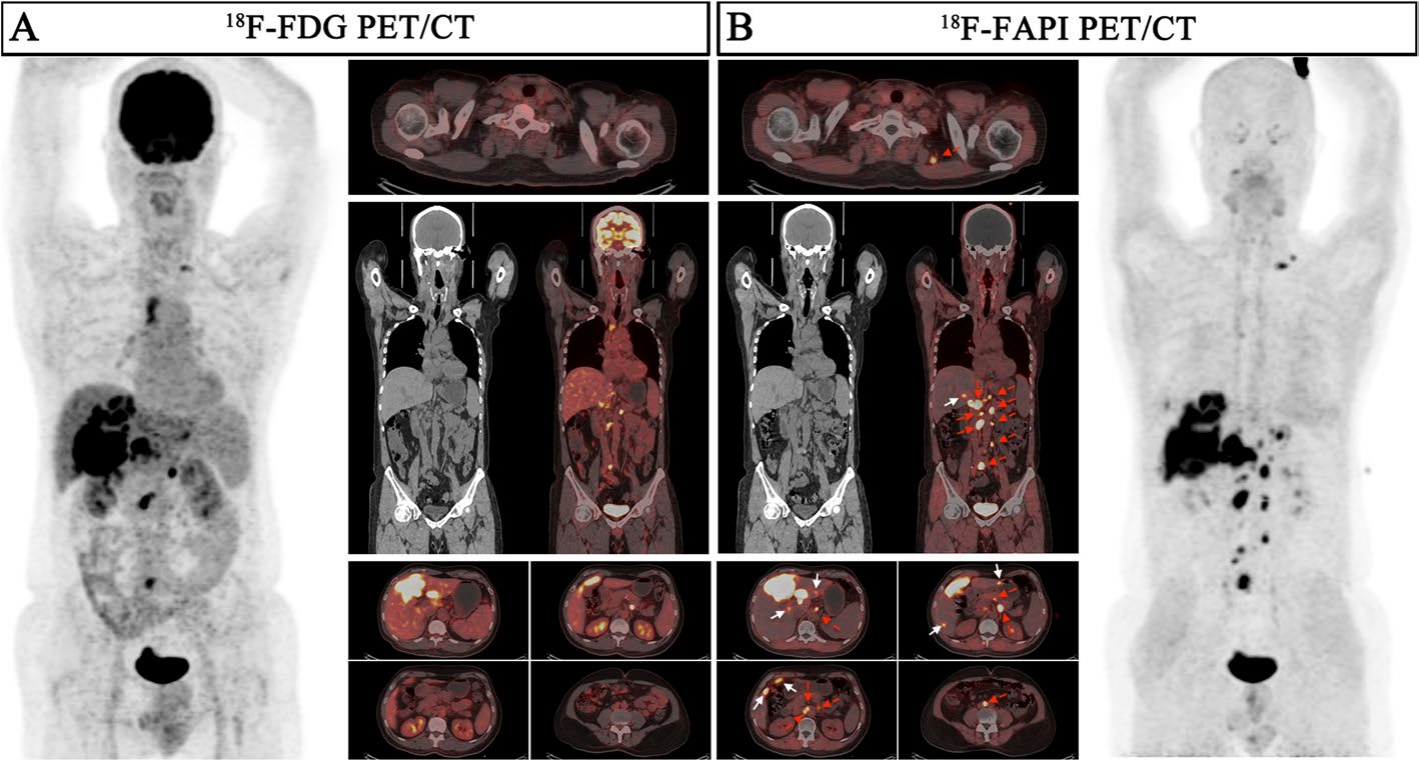
A 62-year-old male patient (Patient No. 13) with HCC (moderately differentiated) was confirmed by biopsy. Compared with ^18^F-FDG PET/CT, ^18^F-FAPI PET/CT revealed more intrahepatic subfoci (white arrow in axial images) and more lymph node metastases (red arrow in axial, coronal images). There was a lymph node in right upper mediastinum, showing low-uptake in ^18^F-FAPI but intense uptake in ^18^F-FDG, final pathological findings confirmed inflammatory

According to the diagnostic criteria for lymph node metastases, 129 lymph nodes lesions in 7 patients were evaluated. The sensitivity of ^18^F-FAPI PET in detecting lymph node metastases was 97.9% (126/129), which was higher than ^18^F-FDG PET [89.1% (115/129), *P* = 0.01]. The TBR of ^18^F-FAPI PET in lymph node metastasis was significantly higher than that in ^18^F-FDG PET (6.3 vs 4.5, *P* < 0.0001), while there was no significant difference in SUV_max_ (7.3 vs 7.6, *P* = 0.7475) (Table 3 and Fig. 2). Sixty-four of 129 (48.8%) lymph node metastasis were greater than 1.0 cm in short diameter, and the detecting rate of ^18^F-FAPI PET and ^18^F-FDG PET for these lymph nodes both were 100%. For lymph node in short diameter (≤ 1.0 cm), the sensitivities of ^18^F-FAPI PET is significantly higher than ^18^F-FDG PET (Fig. 5) [95.4% (62/65) vs. 78.5% (51/65), *P* < 0.0001]. The TBR of ^18^F-FAPI PET in metastasis lymph node (≤ 1.0 cm) were significantly higher than ^18^F-FDG PET (5.0 vs. 3.3, *P* = 0.0016), but there was no significance in SUV_max_ between two agents (Supplementary Fig. 1e & f).

**Table 3:** Comparison of ^18^F-FDG PET/CT and ^18^F-FAPI PET/CT for the extrahepatic lesions

Description of lesions	No. of patients (No. of lesions)	^18^F-FDG PET/CT			^18^F-FAPI PET/CT			*P* value (FDG vs. FAPI)	
		Positive detection of lesions(%)	Median SUV_max_ (range)	Median TBR (range)	Positive detection of lesions(%)	Median SUV_max_ (range)	Median TBR (range)	SUV_max_	TBR
Lymph node metastasis	7(129)	115(89.1%)	7.6(1.2–20.7)	4.5(0.5–17.3)	126(97.9%)	7.3(2.0–21.8)	6.3(1.4–26.5)	0.7475	** < 0.0001**
Head and neck regions	3(16)	13(81.3%)	4.8(1.2–14.7)	2.1(0.5–9.2)	16(100%)	7.7(3.0–13.9)	5.2(1.5–17.4)	**0.0148**	** < 0.0001**
Thoracic regions	2(15)	15(100%)	11.5(3.1–16.4)	7.2(1.3–10.3)	15(100%)	13.1(8.5–21.2)	16.4(4.7–26.5)	**0.0267**	** < 0.0001**
Abdominal regions	7(86)	81(94.2%)	7.95(1.8–20.7)	4.9(0.8–17.3)	83(96.5%)	6.9(2.0–21.8)	5.3(1.4–16.9)	0.0772	0.2818
Pelvic regions	2(12)	6(50%)	2.8(1.7–10.6)	1.3(0.8–6.6)	12(100%)	7.1(3.0–13.8)	7.4(2.3–10.6)	0.1763	**0.0005**
Lymph node size(cm)									
≤ 1	NA(65)	51(78.5%)	5.2(1.2–16.7)	3.3(0.5–13.9)	62(95.4%)	6.2(2.0–21.8)	5.0(1.4–23.1)	0.1253	**0.0016**
> 1	NA(64)	64(100%)	10.55(3.1–20.7)	6.75(1.3–17.3)	64(100%)	9.35(3.1–21.2)	10.15(2.0–26.5)	0.3771	**0.0004**
Distant lesions	9(66)	46(69.7%)	5.0(0.7–25.4)	6.5(0.6–84.7)	42(63.6%)	2.75(0.5–20.4)	7.45(1.7–30.0)	**0.0139**	0.582
Lung	6(24)	7(29.1%)	1.4(0.7–10.2)	6.5(3.5–51.0)	8(37.5%)	1.65(0.5–10.5)	13.0(1.7–28.0)	0.7196	0.0639
Bone	3(24)	24(100%)	11.1(5.2–25.4)	24.15(2.9–84.7)	16(66.7%)	3.0(1.1–20.4)	6.3(2.8–30.0)	**0.0007**	**0.0014**
Peritoneal	1(17)	14(82.4%)	3.9(1.3–7.7)	1.7(0.6–3.3)	17(100%)	4.8(2.3–7.0)	5.3(2.6–7.8)	0.0731	** < 0.0001**
Adrenal gland	1(1)	1(100%)	5.6(NA)	1.9(NA)	1(100%)	4.8(NA)	4.8(NA)	-	-
Total	13(195)	161(82.6%)	6.3(0.7–25.4)	5.1(0.5–84.7)	168(86.2%)	6.4(0.5–21.8)	6.6(1.4–30.0)	0.2538	**0.0007**

Bold fonts indicate significant difference between FDG and FAPI (*P* < 0.05)

^*18*^*F* Fluorine 18, *FDG* fluorodeoxyglucose, *FAPI* fibroblast activation protein inhibitor, *PET/CT* positron emission tomography/computed tomography, *HCC* hepatocellular carcinoma, *SUV*_*max*_ maximum standardized uptake value, *TBR* Tumor-to-background ratio, *NA* Not applicable

### ^18^F-FDG PET and ^18^F-FAPI PET in evaluation of distant metastasis

A total of 66 distant metastatic lesions in 9 patients were confirmed based on the reference standards. There was no statistically significant difference in sensitivity between ^18^F-FAPI PET/CT and ^18^F-FDG PET/CT in detecting distant metastatic lesions [42 (63.6%) vs 46 (69.7%), *P* = 0.58)] (Supplementary Fig. 1g).

Regarding the 3 patients with bone metastasis, ^18^F-FAPI PET had a significant lower sensitivity than ^18^F-FDG PET [66.7% (16/24) vs. 100% (24/24), *P* = 0.004], and ^18^F-FDG PET/CT showed higher SUV_max_ and TBR than ^18^F-FAPI PET/CT in bone metastasis evaluation (median SUV_max_: 11.0 vs 3.0, *P* = 0.0007; median TBR: 24.15 vs 6.3, *P* = 0.0014) (Table 3). Only one patients was diagnosed with peritoneal metastasis (17 lesions, Patient 22 Supplementary Table S1). In contrast to the SUV_max_, the differences between ^18^F-FAPI and ^18^F-FDG imaging were significant quantified by the TBR (median SUV_max_: 4.8 vs 3.9, *P* = 0.0731; median TBR: 5.3 vs 1.7, *P* < 0.0001) (Table 3).

### Changes in staging and therapeutic management

**Fig. 6:**
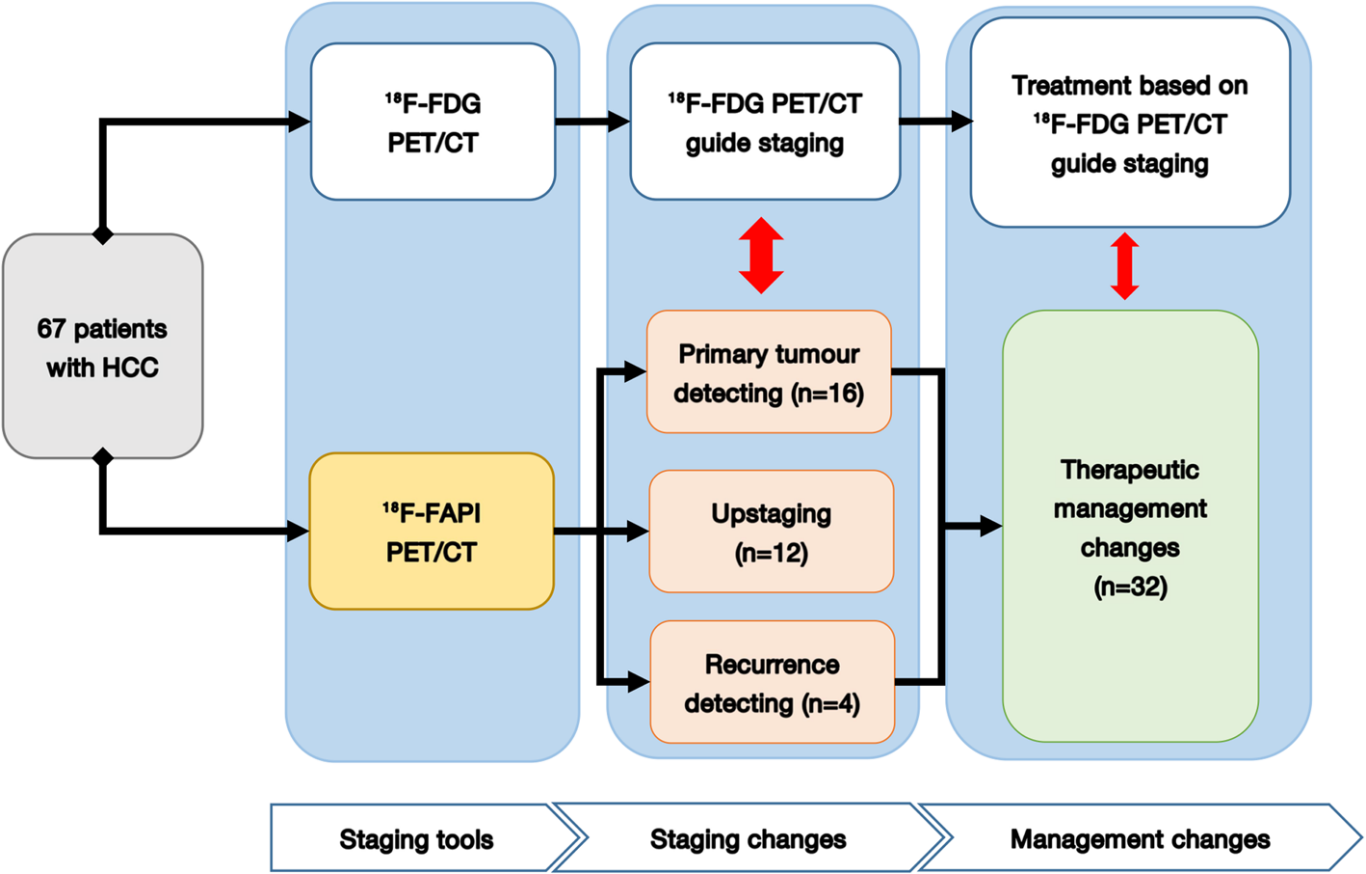
Overview of impact of ^18^F-FAPI PET/CT on staging and therapeutic management in HCC, therapeutic management was altered in 31 of 67 individuals

In the initial assessment of 59 patients, ^18^F-FAPI imaging detected primary HCC tumors in 16 patients with ^18^F-FDG-negative. These patients received the available treatment as early as possible since ^18^F-FAPI detected the primary lesion [11 patients were treated with surgery or ablation; 4 patients with transcatheter arterial chemoembolization (TACE) plus systemic therapy; and 1 patients with TACE plus systemic therapy plus radiotherapy]. With more intrahepatic subfoci revealed by ^18^F-FAPI PET than ^18^F-FDG PET/CT, the TNM staging was upgraded in 12 patients (12/59, 20.3%) (four from IB to II, eight from II to IIIA). As a result, instead of the previously planned surgical treatment, four patient received TACE and systemic chemotherapy, while eight patients received palliative systemic treatment and radiation (Table 4 and Fig. 6).

**Table 4:** Comparison of ^18^F-FDG PET based and ^18^F-FAPI PET based TNM restaging

Patient No	TNM staging (FDG PFT based)	TNM staging (FAPI PFT based)	Additional finding ( ^18^F-FAPI PET)	Staging change	Primary lesion detected
1	**T2**N0M0	**T3**N0M0	Multifocal intrahepatic foci	Up	-
2	**T2**N0M1	**T3**N0M1	Multifocal intrahepatic foci	Up	**-**
4	T2N0M0	T2N0M0	-	None	Yes
6	T1N0M0	T1N0M0	-	None	Yes
7	**T2**N0M0	**T3**N0M0	Multifocal intrahepatic foci	Up	-
10	T1N0M0	T1N0M0	-	None	Yes
15	**T3**N0M0	**T2**N0M0	Multifocal intrahepatic foci	Up	-
16	T1N0M0	T1N0M0	-	None	Yes
18	T1N0M0	T1N0M0	-	None	Yes
19	**T2**N0M0	**T3**N0M0	Multifocal intrahepatic foci	Up	-
21	**T2**N0M0	**T3**N0M0	Multifocal intrahepatic foci	Up	-
23	T1N0M0	T1N0M0	-	None	Yes
25	T1N0M0	T1N0M0	-	None	Yes
26	T1N0M0	T1N0M0	-	None	Yes
27	T2N0M0	T2N0M0	-	None	Yes
28	**T3**N0M1	**T2**N0M1	Multifocal intrahepatic foci	Up	-
29	T2N0M0	T2N0M0	-	None	Yes
30	T3N0M0	T3N0M0	-	None	Yes
31	T1N0M0	T1N0M0	-	None	Yes
36	T2N0M1	T2N0M1	-	None	Yes
44	**T2**N0M1	**T1**N0M1	Multifocal intrahepatic foci	Up	-
51	**T2**N0M0	**T1**N0M0	Multifocal intrahepatic foci	Up	-
55	**T2**N0M0	**T1**N0M0	Multifocal intrahepatic foci	Up	-
56	T1N0M0	T1N0M0	-	None	Yes
60	**T2**N0M0	**T1**N0M0	Multifocal intrahepatic foci	Up	-
63	T1N0M0	T1N0M0	-	None	Yes
65	**T3**N0M0	**T2**N0M0	Multifocal intrahepatic foci	Up	-
66	T1N0M0	T1N0M0	-	None	Yes

Bold fonts indicate the changing segment in TNM stanging after ^18^F-FAPI PET/CT imaging

^*18*^*F* Fluorine 18, *FDG* fluorodeoxyglucose, *FAPI* fibroblast activation protein inhibitor, *PET* positron emission tomography, *TNM* Tumor Node Metastasis

Among the other 8 patients with recurrence, ^18^F-FAPI PET identified ^18^F-FDG-negative locally recurrent tumors in 4 patients (50%) (Table 5), resulting in cancellation of dynamic review and administration of surgery or ablation treatment (Fig. 6).

**Table 5:** Comparison of ^18^F-FDG PET and ^18^F-FAPI PET in post treatment patients

Patient No	Primary treatment	Local recurrence detection		Distant metastasis detection		Additional finding (^18^F-FAPI PET)
		^18^F-FDG PET	^18^F-FAPI PET	^18^F-FDG PET	^18^F-FAPI PET	
3	surgery	-	+	-	-	Local recurrence
32	systemic therapy	+	+	-	-	-
33	TACE	+	+	-	-	-
45	TACE and RFA	-	+	-	-	Local recurrence
48	surgery and systemic therapy	+	+	+	+	-
49	TACE	-	+	+	+	Local recurrence
50	surgery and systemic therapy	-	+	-	-	Local recurrence
58	RFA and systemic therapy	+	+	-	-	-

^*18*^*F* Fluorine 18, *FDG* fluorodeoxyglucose, *FAPI* fibroblast activation protein inhibitor, *PET* positron emission tomography, *TNM* Tumor Node Metastasis, *TACE* transcatheter arterial chemoembolization, *RFA* radiofrequency ablation

## Discussion

Our study have demonstrated ^18^F-FAPI PET/CT plays a complementary role for ^18^F-FDG PET/CT examination in HCC. The results showed ^18^F-FAPI PET/CT is superior to ^18^F-FDG PET/CT in detecting intrahepatic lesions, lymph node metastasis and peritoneal metastasis. In visual analysis, ^18^F-FAPI PET/CT had higher detection rate than ^18^F-FDG PET/CT in intrahepatic lesions (92.2% vs 41.1%, *P* < 0.0001), lymph node metastases (97.9% vs 89.1%, *P* = 0.01). In semiquantitative analysis, the SUV_max_ and TBR of intrahepatic lesions on ^18^F-FAPI PET/CT were both higher than those on ^18^F-FDG PET/CT (all p < 0.01). Although in lymph node and peritoneal metastasis, the SUV_max_ on ^18^F-FAPI PET/CT was not significantly higher than those on ^18^F-FDG PET/CT (all p > 0.05), the TBR on ^18^F-FAPI PET/CT was higher than those on ^18^F-FDG PET/CT (all p < 0.01).

In our study, we demonstrated that ^18^F-FDG is less effective than ^18^F-FAPI in displaying intrahepatic lesions, which was consistent with previous research results [3, 12–14, 18]. The uptake of ^18^F-FDG in malignant tumors largely depends on the presence of facilitated glucose transporters, including type 1 (Glut 1), while Glut 1 is rarely expressed in HCC [7, 21]. Therefore, ^18^F-FDG PET/CT was not recommended for detecting HCC. FAP is overexpressed in CAFs of 90% epithelial carcinomas [10], including HCC, and liver background uptake is low on ^18^F-FAPI PET/CT [18]. Therefore, ^18^F-FAPI PET/CT is superior to ^18^F-FDG PET/CT in detecting intrahepatic lesions. Futhermore, our study compared ^18^F-FDG and ^18^F-FAPI for HCC patients with different T stages, the ability of ^18^F-FAPI PET/CT to display intrahepatic lesions was better than that of ^18^F-FDG PET/CT among patients with T2-T4 stages, and ^18^F-FAPI PET/CT showed significantly higher TBR and similar SUV_max_ in patients with T1 stage. The above results suggest that ^18^F-FAPI PET/CT can improve the ability to detect HCC lesions. In the LI-RADS classification, the diagnosis of HCC lesion with diameter ≤ 2 cm was difficult, and at least two typical imaging manifestations of HCC on ce-CT/ce-MRI/US were required to grade LR-4 [22]. Our results also demonstrated that in addition to LI-RADS, ^18^F-FAPI PET/CT can provide more information by detecting more HCC lesions ≤ 2 cm than ^18^F-FDG PET/CT (88.1% (97/110) vs. 25.5% (28/110), *P* < 0.0001).

Although lymph node metastasis is not common in HCC, it represents the aggressive biological behavior of HCC with poor prognosis [23, 24]. It has been reported that FDG PET/CT is superior to conventional evaluations in detecting occult metastases in patients with invasive HCC. The accuracy of conventional imaging in the diagnosis of lymph node metastasis (short diameter of lymph node ≥ 1 cm] was less than 50% [25]. In visual analysis, our study showed that ^18^F-FAPI PET/CT detected a little more lymph node metastasis than ^18^F-FDG PET/CT. In semi-quantitative analysis, the SUV_max_ of lymph node metastasis on ^18^F-FAPI PET/CT was not significantly higher than that on ^18^F-FDG PET/CT, which could be attributed to HCC that metastasizes to lymph nodes is more aggressive and usually requires more FDG. However, the TBR on ^18^F-FAPI PET/CT was higher than that of ^18^F-FDG PET/CT, which can increase our diagnosis confidence of lymph node metastasis. In additionally, two histology-confirmed inflammatory lymph nodes in paratracheal show ^18^F-FDG false-positive uptake but ^18^F-FAPI negative uptake, intended that ^18^F-FAPI showed the potential ability to differentiate metastatic and nonmetastatic lymph nodes, which was consistent with previous research results [26, 27].

Lung is the most common extrahepatic metastasis site of HCC [28]. Once Lung metastasis occurred in HCC, the patient would be classified into advanced stage and require systemic treatment [1]. In visual analysis and semi-quantitative analysis, ^18^F-FAPI PET/CT and ^18^F-FDG PET/CT showed similar performance on lung metastasis. The background of lung on ^18^F-FAPI PET/CT is as low as that of ^18^F-FDG PET/CT. Besides, in our study, the diameter of more than half of lung metastasis were less than 1 cm, which may lead to less radiopharmaceuticals uptake.

With regard to macrovascular invasion and bone metastasis, the number of lesions detected by ^18^F-FDG PET/CT was higher than that of ^18^F-FAPI PET/CT, and the SUV_max_ and TBR on ^18^F-FDG PET/CT were higher than those on ^18^F-FAPI PET/CT. Several studies have proved that HCC with higher FDG uptake usually displays more aggressive biological behavior [29–31]. The exist of macro and microvascular invasion provide the route for tumor cells to access the portal or systemic circulation, have correlation with the presences of distant metastases [32]. And both macrovascular invasion and distant metastases were the indicator of the aggressiveness of the primary HCC [33, 34]. Although ^18^F-FDG PET/CT is less sensitive in detecting intrahepatic lesions of HCC, it is valuable in detecting macrovascular invasion and bone metastasis. In addition, our study only found one adrenal metastasis lesion which detected both on ^18^F-FAPI PET/CT and ^18^F-FDG PET/CT with equivalent uptake.

In this study, ^18^F-FAPI PET/CT identified more intrahepatic lesions, lymph node metastases and peritoneal metastases than ^18^F-FDG PET/CT in HCC, especially in intrahepatic lesions, and upgraded the T staging in 12 patients. Although ^18^F-FDG PET/CT had advantage in detecting macrovascular invasion and bone metastasis, there was no change in the staging of patients. Therefore, compared with ^18^F-FDG PET/CT, ^18^F-FAPI PET/CT has a greater impact on the initial staging of HCC patients. In addition, in this study, half of the HCC patients (4/8) for restaging found recurrent lesions on ^18^F-FAPI PET/CT, which were negative on ^18^F-FDG PET/CT. Therefore, ^18^F-FAPI PET/CT may demonstrated great value for HCC patients staging and restaging.

Our research also has some limitations. First of all, lymph node metastasis and distant metastasis were followed up by imaging, without pathological results. Secondly, the sample size of distant metastatic lesions is small, the diagnostic value of ^18^F-FAPI PET/CT and ^18^F-FDG PET/CT for HCC in distant metastatic lesions needs to be further explored with a larger sample size. Thirdly, ^18^F-FAPI is not as widely used as ^68^ Ga-FAPI, and more research is needed to confirm its reliability.

## Conclusion

In summary, This prospective study confirmed that ^18^F-FAPI PET/CT is a promising technique in staging of HCC and is complementary to ^18^F-FDG PET/CT. ^18^F-FAPI PET/CT had advantages in detecting intrahepatic lesions, lymph node metastasis and peritoneal metastasis compared to ^18^F-FDG PET/CT, which is helpful to improve the clinical management of HCC patients.

### Supplementary Information

**Additional file 1: Supplementary Fig. 1.** (a & b) Comparison of SUV_max_ and TBR values in different primary tumor staging groups between ^18^F-FDG and ^18^F-FAPI PET. (c & d) Comparison of SUV_max_ and TBR values in different sizes of intrahepatic lesions between ^18^F-FDG and ^18^F-FAPI PET. (e & f) Comparison of SUV_max_ and TBR values in metastatic lymph node with different short diameters (≤ 1 cm or > 1 cm) between ^18^F-FDG and ^18^F-FAPI PET. (g) Compare the performance of ^18^F-FDG and ^18^F-FAPI PET in detecting extrahepatic lesions, involved lymph nodes, lung, bone peritoneal and adrenal gland metastases. ns = no significant; *P < 0.05; **P < 0.01; ***P < 0.001; ****P < 0.0001. **Table S1.** Patient characteristics and ^18^F-FDG/^18^F-FAPI PET/CT imaging findings for the 67 patients

## Data Availability

All the data generated and analyzed during this study are included in our manuscript. The data that support the findings of this study are available from the corresponding author upon reasonable request.

## Abbreviations

CAFs: Cancer-associated fibroblasts
FAPI: Fibroblast activation protein inhibitor
FDG: Fluorodeoxyglucose
HCC: Hepatocellular carcinoma
TBR: Tumor-to-background ratio
PET/CT: Positron emission tomography/computed tomography
SUV_max_: Maximum standardized uptake value

## References

[1] Villanueva A (2019). Hepatocellular carcinoma. N Engl J Med.

[2] Kesler M, Levine C, Hershkovitz D (2019). (68)Ga-PSMA is a novel PET-CT tracer for imaging of hepatocellular carcinoma: A prospective pilot study. J Nucl Med.

[3] Shi X, Xing H, Yang X (2021). Comparison of PET imaging of activated fibroblasts and (18)F-FDG for diagnosis of primary hepatic tumours: a prospective pilot study. Eur J Nucl Med Mol Imaging.

[4] Hirmas N, Leyh C, Sraieb M (2021). (68)Ga-PSMA-11 PET/CT improves tumor detection and impacts management in patients with hepatocellular carcinoma. J Nucl Med.

[5] Park JW, Kim JH, Kim SK (2008). A prospective evaluation of ^18^F-FDG and ^11^C-acetate PET/CT for detection of primary and metastatic hepatocellular carcinoma. J Nucl Med.

[6] Kunikowska J, Cieslak B, Gierej B (2021). [(68) Ga]Ga-Prostate-Specific Membrane Antigen PET/CT: a novel method for imaging patients with hepatocellular carcinoma. Eur J Nucl Med Mol Imaging.

[7] Lee JD, Yang WI, Park YN (2005). Different glucose uptake and glycolytic mechanisms between hepatocellular carcinoma and intrahepatic mass-forming cholangiocarcinoma with increased ^18^F-FDG Uptake. J Nucl Med.

[8] Asman Y, Evenson AR, Even-Sapir E, Shibolet O (2015). ^18^F-fludeoxyglucose positron emission tomography and computed tomography as a prognostic tool before liver transplantation, resection, and loco-ablative therapies for hepatocellular carcinoma. Liver Transpl.

[9] Rettig WJ, Chesa PG, Beresford HR (1986). Differential expression of cell surface antigens and glial fibrillary acidic protein in human astrocytoma subsets. Cancer Res.

[10] Boulter L, Bullock E, Mabruk Z, Brunton VG (2021). The fibrotic and immune microenvironments as targetable drivers of metastasis. Br J Cancer.

[11] Kratochwil C, Flechsig P, Lindner T (2019). (68)Ga-FAPI PET/CT: Tracer Uptake in 28 Different Kinds of Cancer. J Nucl Med.

[12] Guo W, Pang Y, Yao L (2021). Imaging fibroblast activation protein in liver cancer: a single-center post hoc retrospective analysis to compare [(68)Ga]Ga-FAPI-04 PET/CT versus MRI and [(18)F]-FDG PET/CT. Eur J Nucl Med Mol Imaging.

[13] Siripongsatian D, Promteangtrong C, Kunawudhi A (2022). Comparisons of quantitative parameters of ga-68-labelled fibroblast activating protein inhibitor (FAPI) PET/CT and [(18)F]F-FDG PET/CT in patients with liver malignancies. Mol Imaging Biol.

[14] Wang H, Zhu W, Ren S (2021). (68)Ga-FAPI-04 Versus (18)F-FDG PET/CT in the detection of hepatocellular carcinoma. Front Oncol.

[15] Li Y, Lin X, Li Y (2022). Clinical Utility of F-18 Labeled Fibroblast Activation Protein Inhibitor (FAPI) for primary staging in lung adenocarcinoma: a prospective study. Mol Imaging Biol.

[16] Fu L, Huang S, Wu H (2022). Superiority of [(68)Ga]Ga-FAPI-04/[(18)F]FAPI-42 PET/CT to [(18)F]FDG PET/CT in delineating the primary tumor and peritoneal metastasis in initial gastric cancer. Eur Radiol.

[17] Hu K, Wang L, Wu H (2022). [(18)F]FAPI-42 PET imaging in cancer patients: optimal acquisition time, biodistribution, and comparison with [(68)Ga]Ga-FAPI-04. Eur J Nucl Med Mol Imaging.

[18] Zhang J, He Q, Jiang S (2023). [^18^F]FAPI PET/CT in the evaluation of focal liver lesions with [^18^F]FDG non-avidity. Eur J Nucl Med Mol Imaging.

[19] Scialpi M, Palumbo I, Gravante S (2016). FDG PET and split-bolus multi-detector row CT fusion imaging in oncologic patients: preliminary results. Radiology.

[20] Amin MB, Edge SB, Greene FL, et al. eds. AJCC Cancer staging manual, 8th ed. New York: Springer International Publishing: American Joint Commission on Cancer. 2017; 287–294.

[21] Paudyal B, Oriuchi N, Paudyal P (2008). Clinicopathological presentation of varying ^18^F-FDG uptake and expression of glucose transporter 1 and hexokinase II in cases of hepatocellular carcinoma and cholangiocellular carcinoma. Ann Nucl Med.

[22] Chernyak V, Fowler KJ, Kamaya A (2018). Liver imaging reporting and data system (LI-RADS) version 2018: imaging of hepatocellular carcinoma in at-risk patients. Radiology.

[23] Ma J, Chen XQ, Xiang ZL (2022). Identification of a prognostic transcriptome signature for hepatocellular carcinoma with lymph node metastasis. Oxid Med Cell Longev.

[24] Chen X, Lu Y, Shi X (2022). Development and validation of a novel model to predict regional lymph node metastasis in patients with hepatocellular carcinoma. Front Oncol.

[25] Ercolani G, Grazi GL, Ravaioli M (2004). The role of lymphadenectomy for liver tumors: further considerations on the appropriateness of treatment strategy. Ann Surg.

[26] Zhou X, Wang S, Xu X, Meng X, Zhang H, Zhang A (2022). Higher accuracy of [^68^ Ga]Ga-DOTA-FAPI-04 PET/CT comparing with 2-[^18^F]FDG PET/CT in clinical staging of NSCLC. Eur J Nucl Med Mol Imaging.

[27] Wang L, Tang G, Hu K (2022). Comparison of ^68^Ga-FAPI and ^18^F-FDG PET/CT in the evaluation of advanced lung cancer. Radiology.

[28] Uka K, Aikata H, Takaki S (2007). Clinical features and prognosis of patients with extrahepatic metastases from hepatocellular carcinoma. World J Gastroenterol.

[29] Lee JW, Paeng JC, Kang KW (2009). Prediction of tumor recurrence by ^18^F-FDG PET in liver transplantation for hepatocellular carcinoma. J Nucl Med.

[30] Sabate-Llobera A, Mestres-Marti J, Reynes-Llompart G (2021). 2-[(18)F]FDG PET/CT as a Predictor of Microvascular Invasion and High Histological Grade in Patients with Hepatocellular Carcinoma. Cancers (Basel).

[31] Hyun SH, Eo JS, Song B (2018). Preoperative prediction of microvascular invasion of hepatocellular carcinoma using (18)F-FDG PET/CT: a multicenter retrospective cohort study. Eur J Nucl Med Mol Imaging.

[32] Yoneda N, Matsui O, Kobayashi S (2019). Current status of imaging biomarkers predicting the biological nature of hepatocellular carcinoma. Jpn J Radiol.

[33] Lee JW, Hwang SH, Kim HJ, Kim D, Cho A, Yun M (2017). Volumetric parameters on FDG PET can predict early intrahepatic recurrence-free survival in patients with hepatocellular carcinoma after curative surgical resection. Eur J Nucl Med Mol Imaging.

[34] Uchino K, Tateishi R, Shiina S (2011). Hepatocellular carcinoma with extrahepatic metastasis: clinical features and prognostic factors. Cancer.

