# Supplementary material for: Head-to-head comparison of 18F-FAPI and 18F-FDG PET/CT in staging and therapeutic management of hepatocellular carcinoma
Source: Cancer Imaging. 2023 Oct 30;23:106. doi: 10.1186/s40644-023-00626-y (PMC10614420; doi:10.1186/s40644-023-00626-y)
Supplement: Supplementary file 1 — Additional file 1: Supplementary Fig. 1. (a & b) Comparison of SUVmax and TBR values in different primary tumor staging groups between 18F-FDG and 18F-FAPI PET. (c & d) Comparison of SUVmax and TBR values in different sizes of intrahepatic lesions between 18F-FDG and 18F-FAPI PET. (e & f) Comparison of SUVmax and TBR values in metastatic lymph node with different short diameters (≤ 1 cm or > 1 cm) between 18F-FDG and 18F-FAPI PET. (g) Compare the performance of 18F-FDG and 18F-FAPI PET in detecting extrahepatic lesions, involved lymph nodes, lung, bone peritoneal and adrenal gland metastases. ns = no significant; *P < 0.05; **P < 0.01; ***P < 0.001; ****P < 0.0001. Table S1. Patient characteristics and 18F-FDG/18F-FAPI PET/CT imaging findings for the 67 patients [file 40644_2023_626_MOESM1_ESM.docx]

**Supplementary Material**

**[^18^F]FDG/[^18^F]FAPI PET/CT acquisition and imaging**

According to the following procedure, ^18^F-labeled FAP tracers were generated by adding ^18^F^-^ eluent (37-74 GBq) to a solution of DOTA-FAPI (80 nmol) in 2.0 mol/L NaOAc aqueous (1 mL). After being heated at 105 °C for 15 minutes, the reaction mixture was purified using a straightforward solid-phase extraction method followed by cartridge separation. Analysis and quality control of the prepared products were performed in an analytical C18 HPLC column (radiochemical purity>95%).


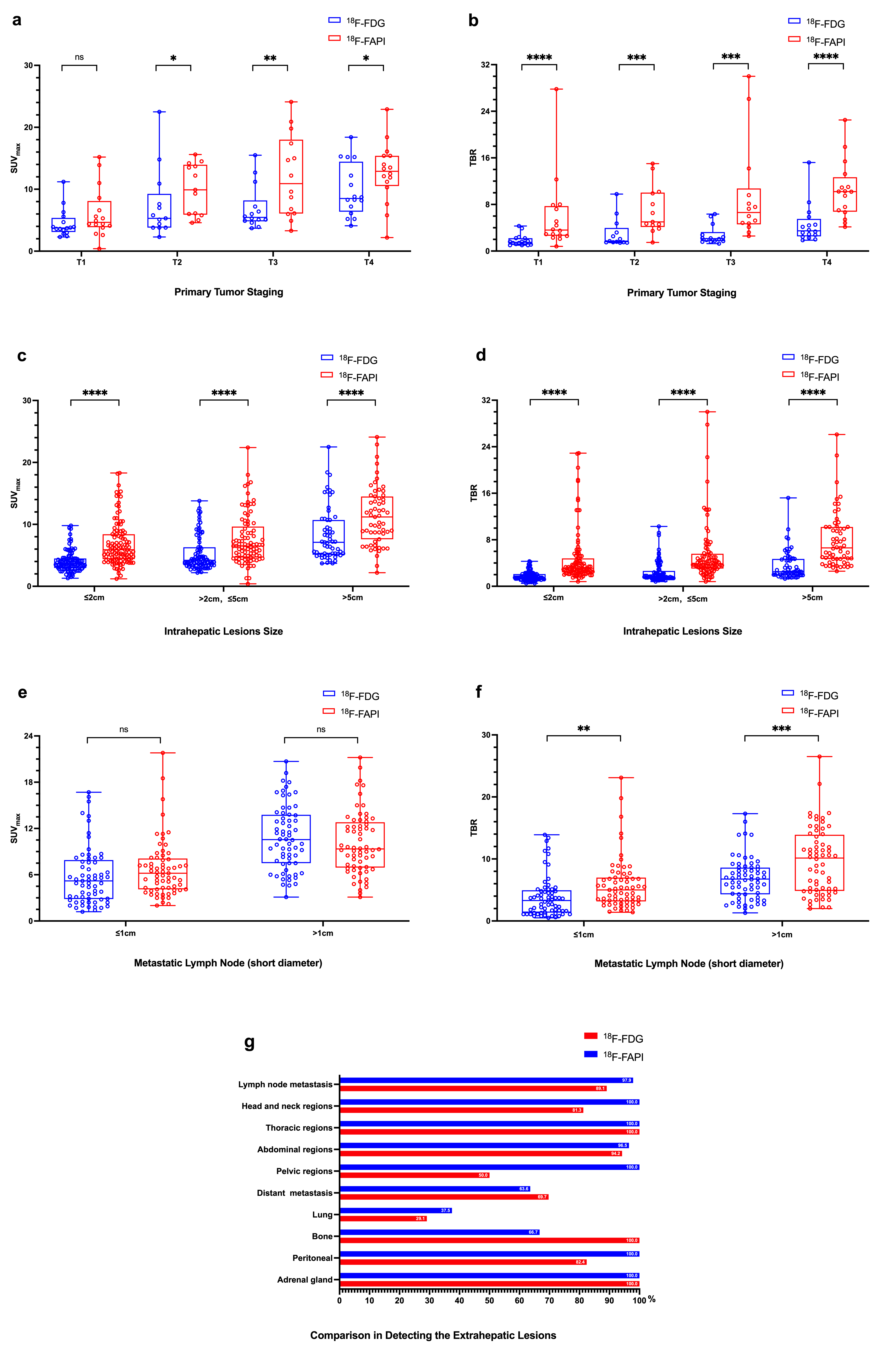


**Supplementary Figure 1. (a&b)** Comparison of SUV_max_ and TBR values in different primary tumor staging groups between ^18^F-FDG and ^18^F-FAPI PET. **(c&d)** Comparison of SUV_max_ and TBR values in different sizes of intrahepatic lesions between ^18^F-FDG and ^18^F-FAPI PET. **(e&f)** Comparison of SUV_max_ and TBR values in metastatic lymph node with different short diameters (≤1cm or >1cm) between ^18^F-FDG and ^18^F-FAPI PET. **(g)** Compare the performance of ^18^F-FDG and ^18^F-FAPI PET in detecting extrahepatic lesions, involved lymph nodes, lung, bone peritoneal and adrenal gland metastases. ns = no significant; **P* < 0.05; ***P* < 0.01; ****P* < 0.001; *****P* < 0.0001

Table S1. Patient characteristics and ^18^F-FDG/^18^F-FAPI PET/CT imaging findings for the 67 patients

| Patient No. | Age | Sex | primary stageing /recurrence | Intrahepatic lesions detection based on ce-CT/ce-MRI | ^18^F-FDG PET/CT | | |  | ^18^F-FAPI PET/CT | | | Lymph node metastasis | Distant metastasis |
| --- | --- | --- | --- | --- | --- | --- | --- | --- | --- | --- | --- | --- | --- |
|  |  |  |  |  | Positive detection of lesions | Median SUVmax (range) | Median TBR (range) |  | Positive detection of lesions | Median SUVmax (range) | Median TBR (range) |  |  |
| 1 | 68 | M | primary staging | 2 | 1 | 4.65(3.3-6) | 1.7(1.2-2.2) |  | 2 | 10.4(5.8-15) | 20.8(11.6-30) |  |  |
| 2 | 68 | M | primary staging | 16 | 2 | 3.75(1.3-6.1) | 1.4(0.5-2.3) |  | 15 | 7.45(4.4-17.4) | 3.2(1.9-7.6) |  | Lung |
| 3 | 70 | M | Recurrence | 1 | 0 | 3.4 | 1.4 |  | 1 | 2.9 | 4.1 |  |  |
| 4 | 67 | M | primary staging | 1 | 0 | 5.3 | 1.7 |  | 1 | 13.7 | 6.5 |  |  |
| 5 | 65 | M | primary staging | 1 | 1 | 11.2 | 4.3 |  | 1 | 13.9 | 27.8 |  |  |
| 6 | 56 | M | primary staging | 1 | 0 | 3.4 | 1.6 |  | 1 | 15.2 | 2.6 |  |  |
| 7 | 61 | M | primary staging | 2 | 0 | 4.6(3.9-5.3) | 1.5(1.3-1.7) |  | 2 | 5.75(5.5-6) | 5.75(5.5-6) |  |  |
| 8 | 63 | F | primary staging | 13 | 11 | 4.8(2.8-12.7) | 2.4(1.4-6.4) |  | 13 | 12.1(6-20.9) | 15.1(7.5-26.1) | + | Bone |
| 9 | 46 | M | primary staging | 1 | 1 | 7.8 | 3.9 |  | 1 | 5.6 | 8.0 |  |  |
| 10 | 48 | M | primary staging | 1 | 0 | 3.9 | 1.7 |  | 1 | 8.6 | 12.3 |  |  |
| 11 | 62 | M | primary staging | 1 | 1 | 7.6 | 2.5 |  | 1 | 12.2 | 10.2 |  |  |
| 12 | 66 | M | primary staging | 1 | 1 | 14.8 | 6.4 |  | 1 | 15.6 | 14.2 |  |  |
| 13 | 62 | M | primary staging | 18 | 8 | 4.9(2.3-12.2) | 1.8(0.9-4.5) |  | 18 | 5.45(2.9-18) | 5.45(2.9-18) | + |  |
| 14 | 61 | M | primary staging | 11 | 9 | 5.8(3.1-9.5) | 2.9(1.6-4.8) |  | 11 | 10.2(5.6-18.4) | 5.7(3.1-10.2) |  | Lung |
| 15 | 54 | M | primary staging | 5 | 1 | 4.1(3.8-5.1) | 1.3(1.2-1.6) |  | 2 | 1.3(1.2-3.3) | 1.6(1.5-4.1) |  |  |
| 16 | 57 | M | primary staging | 1 | 0 | 3.6 | 1.2 |  | 0 | 0.4 | 0.8 |  |  |
| 17 | 67 | M | primary staging | 1 | 0 | 4.1 | 1.9 |  | 1 | 2.2 | 11.0 |  |  |
| 18 | 64 | M | primary staging | 1 | 0 | 3.8 | 1.4 |  | 1 | 3.8 | 3.5 |  |  |
| 19 | 68 | F | primary staging | 9 | 1 | 2.9(2.5-4.6) | 1.4(1.2-2.2) |  | 7 | 8.8(5.9-14.7) | 2.8(1.9-4.7) |  |  |
| 20 | 74 | M | primary staging | 1 | 1 | 6.3 | 2.2 |  | 1 | 5.3 | 2.9 |  |  |
| 21 | 83 | M | primary staging | 8 | 1 | 3.7(2.9-6.4) | 1.6(1.3-2.8) |  | 5 | 10.8(6.5-19.8) | 2.6(1.6-4.8) |  |  |
| 22 | 48 | F | primary staging | 1 | 1 | 22.5 | 9.8 |  | 1 | 13.5 | 15.0 | + | Peritoneal |
| 23 | 47 | M | primary staging | 1 | 0 | 2.8 | 1.0 |  | 1 | 2.8 | 2.3 |  |  |
| 24 | 72 | M | primary staging | 3 | 3 | 8.7(8.5-11.2) | 3.6(3.5-4.7) |  | 3 | 22.4(16.2-24.1) | 13.2(9.5-14.2) |  |  |
| 25 | 53 | F | primary staging | 1 | 0 | 3.9 | 1.6 |  | 1 | 4.9 | 2.1 |  |  |
| 26 | 51 | M | primary staging | 1 | 0 | 3.6 | 1.4 |  | 1 | 4.3 | 7.2 |  |  |
| 27 | 41 | M | primary staging | 1 | 0 | 5.1 | 1.5 |  | 1 | 6.1 | 4.7 |  |  |
| 28 | 54 | M | primary staging | 3 | 1 | 4.2(3.4-5.1) | 1.4(1.2-1.8) |  | 3 | 6.4(4-6.8) | 4.9(3.1-5.2) |  | Adrenal gland, Bone |
| 29 | 67 | M | primary staging | 2 | 0 | 3.75(3.7-3.8) | 1.5(1.48-1.52) |  | 2 | 3.35(2.1-4.6) | 3.35(2.1-4.6) |  |  |
| 30 | 67 | M | primary staging | 2 | 0 | 3.65(3.6-3.7) | 1.25(1.2-1.3) |  | 2 | 6(5.9-6.1) | 3.2(3.1-3.2) |  |  |
| 31 | 51 | M | primary staging | 1 | 0 | 2.3 | 1.0 |  | 1 | 11.0 | 7.9 |  |  |
| 32 | 56 | M | Recurrence | 2 | 2 | 6.55(3.8-9.3) | 3.6(2.1-5.2) |  | 2 | 12.85(8.9-16.8) | 9.9(6.8-12.9) |  |  |
| 33 | 56 | M | Recurrence | 9 | 1 | 2.8(1.7-9.4) | 1.1(0.7-3.8) |  | 6 | 8.4(1.7-11.9) | 4.0(0.8-5.7) |  |  |
| 34 | 45 | M | primary staging | 5 | 3 | 5.9(1.7-15.2) | 2.3(0.7-5.8) |  | 3 | 3.9(2.8-13.3) | 2.1(1.5-7.0) |  |  |
| 35 | 53 | M | primary staging | 2 | 2 | 9.4(8.1-10.7) | 2.9(2.5-3.3) |  | 2 | 12.5(8.9-16.1) | 13.9(9.9-17.9) |  |  |
| 36 | 62 | M | primary staging | 1 | 0 | 3.8 | 1.5 |  | 1 | 14.5 | 8.1 |  | Lung |
| 37 | 50 | M | primary staging | 1 | 1 | 8.2 | 3.2 |  | 1 | 7.6 | 9.5 |  |  |
| 38 | 56 | M | primary staging | 1 | 1 | 7.0 | 3.2 |  | 1 | 14.2 | 3.5 |  |  |
| 39 | 79 | M | primary staging | 2 | 2 | 7.25(7.2-7.3) | 2.4(2.4-2.43) |  | 2 | 10.8(9.4-12.2) | 7.2(6.3-8.1) | + |  |
| 40 | 53 | F | primary staging | 3 | 2 | 15.5(4.6-16) | 6.0(1.8-6.2) |  | 3 | 5.7(3.3-9.6) | 5.7(3.3-9.6) | + |  |
| 41 | 64 | F | primary staging | 5 | 4 | 7.5(3.3-8.4) | 3.8(1.7-4.2) |  | 5 | 6.4(4.1-12.1) | 4.0(2.6-7.6) |  |  |
| 42 | 54 | F | primary staging | 8 | 1 | 3.4(1.5-8.7) | 1.1(0.5-2.9) |  | 6 | 5.65(3.6-22.9) | 2.7(1.7-10.9) |  |  |
| 43 | 62 | M | primary staging | 14 | 6 | 5.85(4.2-8.3) | 2.0(1.4-2.8) |  | 14 | 8.95(6.1-12.5) | 3.4(2.3-4.8) | + |  |
| 44 | 64 | M | primary staging | 3 | 1 | 3.1(3-4.4) | 1.0(1.0-1.4) |  | 2 | 5.4(4.6-5.8) | 1.4(1.2-1.5) |  |  |
| 45 | 51 | M | Recurrence | 1 | 0 | 4.6 | 1.8 |  | 1 | 4.9 | 2.6 |  |  |
| 46 | 49 | M | primary staging | 15 | 13 | 10.7(3.2-18.4) | 4.9(1.5-8.4) |  | 15 | 6.2(3.3-15.4) | 4.1(2.2-10.3) |  | Lung, Bone |
| 47 | 32 | F | primary staging | 14 | 4 | 4.55(3.8-6.1) | 1.8(1.5-2.4) |  | 13 | 5.25(3.6-14) | 2.5(1.7-6.7) |  |  |
| 48 | 59 | M | Recurrence | 7 | 2 | 3.7(3-11.8) | 1.5(1.3-4.9) |  | 7 | 6.5(4.5-10.5) | 3.1(2.1-5.0) | + | Lung |
| 49 | 71 | M | Recurrence | 2 | 0 | 3(2.5-3.5) | 1.0(0.8-1.2) |  | 2 | 6.75(6.2-7.3) | 6.1(5.6-6.6) |  | Lung |
| 50 | 57 | M | Recurrence | 1 | 0 | 2.4 | 1.0 |  | 1 | 9.0 | 6.0 |  |  |
| 51 | 41 | M | primary staging | 6 | 1 | 3.4(3.2-5.8) | 1.2(1.1-2.1) |  | 6 | 6.7(3-9.3) | 3.2(1.4-4.4) |  |  |
| 52 | 42 | M | primary staging | 1 | 1 | 5.2 | 2.3 |  | 1 | 5.8 | 4.1 |  |  |
| 53 | 40 | F | primary staging | 4 | 4 | 9.75(8.8-15.2) | 9.8(8.8-15.2) |  | 4 | 9.65(4.7-13.5) | 16.1(7.8-22.5) |  |  |
| 54 | 68 | M | primary staging | 1 | 1 | 5.6 | 2.2 |  | 1 | 6.6 | 5.1 |  |  |
| 55 | 56 | M | primary staging | 2 | 1 | 3.25(2.7-3.8) | 1.4(1.1-1.6) |  | 2 | 4.35(3.7-5) | 3.3(2.8-3.8) |  |  |
| 56 | 59 | M | primary staging | 1 | 0 | 3.7 | 1.4 |  | 1 | 4.4 | 3.7 |  |  |
| 57 | 50 | M | primary staging | 13 | 0 | 3.9(2.9-4.7) | 1.6(1.2-2.0) |  | 13 | 6.6(4.3-8.1) | 3.5(2.3-4.3) |  |  |
| 58 | 36 | M | Recurrence | 1 | 0 | 3.7 | 1.5 |  | 1 | 11.4 | 5.2 |  |  |
| 59 | 68 | M | primary staging | 5 | 3 | 4.8(2.8-15.3) | 2.1(1.2-2.0) |  | 5 | 4.9(3.7-11.2) | 4.5(3.4-10.2) |  |  |
| 60 | 68 | M | primary staging | 5 | 1 | 2.5(2.2-2.9) | 1.6(1.4-1.8) |  | 5 | 4.1(3.9-6) | 3.4(3.3-5.0) |  |  |
| 61 | 49 | M | primary staging | 4 | 2 | 4.4(2-7.2) | 2.2(1.0-3.6) |  | 3 | 5.55(3.5-10.3) | 2.9(1.8-5.4) |  |  |
| 62 | 81 | M | primary staging | 1 | 1 | 4.7 | 2.4 |  | 1 | 4.4 | 4.4 |  |  |
| 63 | 41 | M | primary staging | 1 | 0 | 3.0 | 1.1 |  | 1 | 4.1 | 2.7 |  |  |
| 64 | 56 | M | primary staging | 1 | 0 | 5.2 | 2.0 |  | 1 | 11.9 | 13.2 |  |  |
| 65 | 77 | M | primary staging | 2 | 1 | 4.3(3.7-4.9) | 1.7(1.4-1.9) |  | 2 | 6.45(4.2-8.7) | 5.4(3.5-7.25) |  |  |
| 66 | 55 | M | primary staging | 1 | 0 | 2.4 | 1.0 |  | 1 | 2.6 | 2.9 |  |  |
| 67 | 52 | F | primary staging | 1 | 1 | 10.9 | 4.7 |  | 1 | 9.9 | 9.9 |  |  |
